# Predictive value of the preoperative C-reactive protein-to-albumin ratio for surgical site infection after percutaneous kyphoplasty: a single-center retrospective study

**DOI:** 10.3389/fcimb.2025.1565468

**Published:** 2025-04-17

**Authors:** Shuai Tang, Wenhua Gong, Xiaocui Han, Shuo Han, Hao Zhang, Zheng Lian

**Affiliations:** ^1^ Department of Spinal Surgery, The Affiliated Hospital of Qingdao University, Qingdao, China; ^2^ Department of Pathology, The Affiliated Hospital of Qingdao University, Qingdao, China

**Keywords:** C-reactive protein-to-albumin ratio, lumbar vertebral compression fracture, thoracic vertebral compression fracture, percutaneous kyphoplasty, surgical site infection, risk factor

## Abstract

**Objective:**

This study aimed to investigate the risk factors for surgical site infection (SSI) after percutaneous kyphoplasty (PKP) and evaluate the application value of the preoperative C-reactive protein (CRP)-to-albumin ratio (CAR) in predicting SSI.

**Methods:**

This study retrospectively enrolled 329 patients with thoracolumbar compression fractures who underwent PKP in the Affiliated Hospital of Qingdao University from January 2019 to June 2024. The demographic information, surgery-related data and laboratory examination results of the patients were collected. According to these results, the patients were divided into SSI and non-SSI groups, and the results were compared and analyzed. The receiver operating characteristic curve was used to determine the optimal cutoff value of preoperative CAR for predicting SSI, and binary logistic regression analysis was employed to evaluate the predictive value of CAR for SSI. The risk factors of SSI in the thoracolumbar subgroup were further explored.

**Results:**

The study enrolled a total of 329 patients, and SSI occurred in 29 (8.81%). The optimal cut-off value of CAR was 0.1213, and the area under the curve was 0.808 (P < 0. 001). The results showed that SSI rates were related to the surgical site, and the SSI rate in the lumbar spine was higher than that in the thoracic spine. The SSI group had a longer surgical duration and more operated segments. The levels of preoperative CRP, CAR, procalcitonin and erythrocyte sedimentation rate (ESR) were higher; however, serum albumin levels were lower. More patients had CAR ≥0.1213 (75.86% vs 25.33%) and white blood cell (WBC) >10*10^9^ (27.59% vs 10.00%). In addition, no significant differences were found by the other demographic data and laboratory examinations between the two groups. In the binary logistic regression analysis, preoperative CAR was an independent risk factor for post-PKP SSI, and the SSI risk increased by 7.464 times in patients with CAR ≥0.1213. The number of operated segments, surgical duration, and ESR were also independent risk factors for SSI, whereas serum albumin is a protective factor.

**Conclusion:**

Preoperative CAR is an effective predictor of post-PKP SSI, which can be used for clinical prevention and reduction of SSI risk.

## 1 Introduction

As the global population aging accelerates and human lifestyles change, the increasing incidence of age-related osteoporosis and vertebral compression fractures becomes a severe public health challenge. According to the epidemiological survey, the incidence of thoracolumbar vertebral compression fractures in older people in China is as high as 14.2% (Oei et al., 2018).

Percutaneous kyphoplasty (PKP) is a widely performed treatment for thoracolumbar compression fractures, owing to its simplicity, safety, and relatively high cost-effectiveness. By quickly reinforcing and stabilizing the fractured vertebra, PKP helps restore its weight-bearing function, alleviate pain, and significantly improve the quality of life (Boonen et al., 2011; Papanastassiou et al., 2012; Zhu et al., 2022).

However, the widespread adoption of a surgical technique is bound to draw significant attention from doctors and patients to its complications. A literature review revealed a low risk of surgical site infection (SSI) in patients who underwent PKP, with an incidence of 0.36% (Park et al., 2018). However, the consequences of post-PKP SSI are often unpredictable and catastrophic, which has a serious effect on the patient and his/her family and even requires a second operation, which is associated with serious trauma and financial burden to the patient.

Identifying the associated factors is the most economical way to prevent or minimize such adverse events, allowing for the stratification of individual risk and the implementation of targeted preventive actions. At present, an increasing number of studies worldwide emphasize that clinical judgment alone cannot be relied on to develop more accurate risk prediction tools because of its subjectivity and lagging performance or signs (Ziegler et al., 2022). Unfortunately, currently, no well-established risk prediction system has been specifically designed to assess the occurrence of post-PKP SSI.

The C-reactive protein-to-albumin ratio (CAR) has been proven to have value in predicting clinical outcomes or postoperative complications in various diseases, such as ST-segment elevation myocardial infarction (Askin et al., 2020), Guillain–Barre syndrome (Ning et al., 2021), gastrointestinal cancer (Goulart et al., 2018; Alkurt et al., 2022), urothelial cancer (Uchimoto et al., 2024), hepatocellular carcinoma (Ren et al., 2019; Mai et al., 2024), lung cancer (Jia-Min et al., 2022), joint or musculoskeletal disease (Li et al., 2017; Capkin et al., 2021; Pamukcu and Duran, 2021; Wu et al., 2023), and so on.

To our best knowledge, no study has investigated the role of CAR in predicting post-PKP SSI. Accordingly, this study aimed to determine the risk factors for the occurrence of post-PKP SSI for thoracolumbar compression fractures and evaluate the utility of CAR in predicting SSI using binary logistic regression analysis with adjustments for various confounders.

## 2 Materials and methods

### 2.1 Patient selection

We conducted a retrospective cross-sectional study based on data from 362 patients who underwent PKP for thoracic or lumbar vertebral compression fractures between January 2019 and June 2024 in the Affiliated Hospital of Qingdao University. The study was approved by the Ethics Committee of the Affiliated Hospital of Qingdao University. Exclusion criteria were patients with missing clinical indicators (n = 18), missing laboratory examinations (n = 10), and patients with metastatic vertebral body tumor (n = 5). A total of 329 patients were included in our analysis, and patients with active infections or those receiving antibiotics within 7 days preoperatively were explicitly excluded. All procedures followed institutional protocols for preoperative skin disinfection, intraoperative aseptic techniques, and postoperative wound care to standardize perioperative management. Based on the clinical signs, blood biochemical, and microbiological examinations, all patients enrolled in the study were divided into two groups: the SSI and the non-SSI groups. According to the sample size formula of Logistic regression, only 60 samples were needed to detect the effect size of OR=7.464 (α = 0.05, power = 80%), and the current sample size (329 cases) was far beyond the demand.

### 2.2 Confirmation of SSI

The diagnosis of SSI was based on the Centers for Disease Control and Prevention Guideline for the Prevention of Surgical Site Infection (2017 version) (Berríos-Torres et al., 2017). SSI was defined as infection that occurred in the surgical incision and surrounding tissues within 30 days of surgery. SSI could be further divided into superficial SSI, deep SSI, and organ or interstitial space infection. Superficial SSI was defined as skin and subcutaneous tissue infection occurring within 30 days after surgery. The clinical signs included local redness, swelling, heat, pain or purulent discharge of the incision. Positive bacterial culture was the main basis for diagnosis. Deep SSI referred to infection that occurred within 1 year after implant surgery, involving deep tissues of the incision (such as deep fascia and muscle). Clinical signs included drainage of pus from deep part of the incision or fever ≥38°C, and positive bacterial culture also supported the diagnosis. Organ or lacunar infection was defined as occurring within 30 days postoperatively without implants or within 1 year after surgery with implants. The infection involved organs or lacunar spaces outside the surgical incision. Clinical signs may include drainage of pus or evidence of infection on imaging examination. The diagnosis of SSI not only depended on clinical manifestations, but also included microbiological examination, among which positive bacterial culture was an important basis for the diagnosis of SSI. Based on the above criteria, we screened for cases of SSI after PKP.

### 2.3 Demographic data

All patients’ age, sex, body mass index (BMI), hypertension, diabetes mellitus, tobacco use, drinking history, surgical site, surgical duration, the number of operated segments, and preoperative body temperature were recorded and collected. BMI was calculated as follows: BMI = weight/height2 (kg/m2). Patients who had smoked or consumed alcohol at least once per month during the past 6 months before the index operation were considered current smokers or drinkers (Liao et al., 2018).

### 2.4 Laboratory examinations

Preoperative biochemical data of venous blood samples were collected. Preoperative laboratory biomarkers included preoperative CRP, serum albumin, procalcitonin, erythrocyte sedimentation rate (ESR), white blood cell (WBC), neutrophil, lymphocyte, red blood cell (RBC), hemoglobin, platelet, and fasting blood glucose (FBG). If a patient had multiple preoperative measurements of a biochemical biomarker, the one closest to the surgery was selected for analysis to minimize potential time-dependent confounding effects.

### 2.5 Statistical analysis

All analyses were performed using IBM SPSS Statistics for Windows version 27.0 (IBM Corp., Armonk, NY USA). The normality of the distribution of the measurement data was evaluated by the Kolmogorov–Smirnov test. Continuous normally distributed variables were expressed as mean ± standard deviation, and categorical variables were expressed as percentage (%). The clinical continuous variables were compared between the cohorts using Student’s t-test. The clinical categorical variables were assessed by chi-square test.

The receiver operating characteristic (ROC) curve was constructed to determine the optimal cut-off value of CAR for predicting the possibility of SSI, when the specificity plus sensitivity was maximum. The area under the curve (AUC) was calculated to evaluate the discrimination ability. According to the optimal cut-off value, patients were divided into high and low CAR groups to form a set of categorical variables for the chi-square test. In order to evaluate the stability of the model and the confidence interval of parameter estimates, the Bootstrap resampling method was used to internally validate the prediction model (Harrell, 2001).

The factors with P-values <0.05 selected in Student’s t-test and chi-square test were further incorporated into the dichotomous logistic regression analyses (Iasonos et al., 2008). To determine the significant and independent risk factors associated with SSI, dichotomous logistic regression analysis (stepwise regression analysis method) was also performed to calculate odds ratios and 95% confidence intervals. A P-value <0.05 was considered statistically significant.

## 3 Results

### 3.1 Demographic data

**Figure 1:**
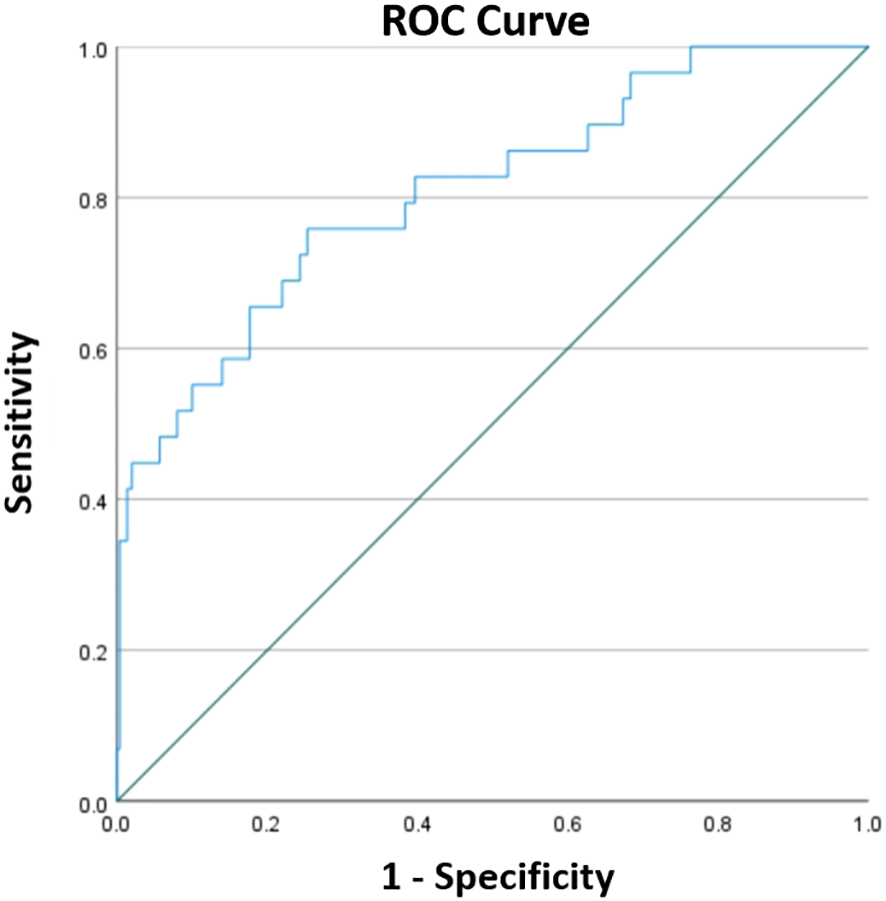
The ROC curve constructed to determine the optimal cut-off for CAR. The optimal cut-off was 0.1213, corresponding to a sensitivity of 0.759 and a specificity of 0.747, and the area under the curve was 0.808 (95% CI, 0.718–0.898; P < 0.001).

In total, 329 patients (mean age, 68.73 ± 6.72 years; 90 male and 239 female) were enrolled in the study. Of these patients, 137 (41.64%) had hypertension, 62 (18.84%) had diabetes mellitus, 16 (4.86%) had a history of tobacco use, and 14 (4.26%) had a history of drinking. Among them, 109 (33.13%) patients underwent PKP for lumbar compression fractures and 220 (66.87%) for thoracic vertebrae. The mean BMI was 24.48 ± 3.55 kg/m2. The mean surgical duration was 53.42 ± 24.14 minutes, and the mean number of operated segments was 1.15 ± 0.38. The mean CRP was 3.89 ± 3.85 mg/L, and the mean serum albumin was 39.16 ± 3.39 g/L. As shown in **Figure 1**, the optimal cut-off value of CAR was 0.1213, corresponding to a sensitivity of 0.759 and a specificity of 0.747, and the area under the curve was 0.808 (95% CI, 0.718–0.898; P <.001). In this study, 98 patients with a CAR of 0.1213 or higher. Additional demographic data and blood biochemical data of the enrolled patients are presented in **Table 1**.

**Table 1:** Demographic data of all patients.

Variables	All Patients (n=329)
Sex (n,%)	
Male	90(27.36)
Female	239(72.64)
Age (yr)	68.73 ± 6.72
BMI (kg/m^2^)	24.48 ± 3.55
Hypertension (n,%)	137(41.64)
Diabetes mellitus (n,%)	62(18.84)
Tobacco use (n,%)	16(4.86)
Drinking history (n,%)	14(4.26)
Surgical Site (n,%)	
Lumbar spine	109(33.13)
Thoracic vertebrae	220(66.87)
Surgical duration (minutes)	53.42 ± 24.14
The number of operated segments	1.15 ± 0.38
Preoperative body temperature ≥ 37.2°C (n,%)	37(11.25)
Preoperative blood biochemical indicators	
CRP (mg/L)	3.89 ± 3.85
Serum albumin (g/L)	39.16 ± 3.39
CAR (mg/g)	0.103 ± 0.113
CAR ≥ 0.1213 (n,%)	98(29.79)
Procalcitonin (ng/mL)	0.043 ± 0.025
ESR (mm/h)	20.71 ± 13.42
WBC > 10*10^9^/L (n,%)	38(11.55)
Neutrophil > 6.3*10^9^/L (n,%)	42(12.77)
Lymphocyte < 1.1*10^9^/L (n,%)	52(15.81)
RBC < Lower limit (n,%)	47(14.29)
Hemoglobin < Lower limit (n,%)	54(16.41)
Platelet > 220*10^9^ (n,%)	185(56.23)
FBG (mmol/L)	5.87 ± 1.63

RBC, red blood cell, reference range: females, 3.5-5.0 × 10^12^/L; males, 4.0-5.5 × 10^12^/L; Hemoglobin, reference range: females, 110 to 150 g/L; males, 120 to 160 g/L.

### 3.2 Comparison between the SSI and Non-SSI groups

SSI was observed in 29 (8.81%) patients, and 300 (91.19%) patients had no SSI (non-SSI group).

In the SSI group, 14 (48.28%) patients had hypertension, 4 (13.79%) had diabetes mellitus, 1 (3.45%) had a history of tobacco use, and 2 (6.90%) had a history of drinking. The incidence of SSI was related to the surgical site. In the SSI group, there were 15 (51.72%) patients with postoperative infection of lumbar compression fractures and 14 (48.28%) patients with postoperative infection of thoracic vertebrae. Moreover, the SSI group had longer surgical duration (89.83 ± 36.97 vs 49.90 ± 19.25 min, P < 0.001) and more surgical segments (1.59 ± 0.57 vs 1.11 ± 0.33, P < 0.001). In the preoperative blood biochemical indicators, the SSI group had higher levels of CRP (9.50 ± 7.00 vs 3.35 ± 2.89 mg/L, P < 0.001), CAR (0.283 ± 0.231 vs 0.085 ± 0.075, P < 0.001), procalcitonin (0.079 ± 0.044 vs 0.039 ± 0.019 ng/mL, P < 0.001), ESR (38.76 ± 25.95 vs 18.96 ± 9.99 mm/h, P < 0.001), and lower levels of serum albumin (35.37 ± 4.65 vs 39.53 ± 3.01 g/L, P < 0.001), as well as a larger proportion of patients with CAR ≥0.1213 (75.86% vs 25.33%) and WBC > 10*109 (27.59% vs 10.00%). In addition, there were no significant differences in sex, age, BMI, hypertension, diabetes mellitus, tobacco use, drinking history, preoperative body temperature, and neutrophil, lymphocyte, RBC, and platelet count; hemoglobin levels; FBG between the two groups (**Table 2**).

**Table 2:** Comparison between SSI group and non-SSI group.

Variables	SSI Group (n=29)	Non-SSI Group (n=300)	P-value
Sex (n,%)			0.367
Male	10(34.48)	80(26.67)	
Female	19(65.52)	220(73.33)	
Age (yr)	70.52 ± 6.31	68.56 ± 6.74	0.134
BMI (kg/m^2^)	24.86 ± 2.38	24.44 ± 3.64	0.398
Hypertension (n,%)	14(48.28)	123(41.00)	0.448
Diabetes mellitus (n,%)	4(13.79)	58(19.33)	0.466
Tobacco use (n,%)	1(3.45)	15(5.00)	0.711
Drinking history (n,%)	2(6.90)	12(4.00)	0.461
Surgical Site (n,%)			0.026*
Lumbar spine	15(51.72)	94(31.33)	
Thoracic vertebrae	14(48.28)	206(68.67)	
Surgical duration (minutes)	89.83 ± 36.97	49.90 ± 19.25	<0.001*
The number of operated segments	1.59 ± 0.57	1.11 ± 0.33	<0.001*
Preoperative body temperature ≥ 37.2°C (n,%)	6(20.69)	31(10.33)	0.092
Preoperative blood biochemical indicators			
CRP (mg/L)	9.50 ± 7.00	3.35 ± 2.89	<0.001*
Serum albumin (g/L)	35.37 ± 4.65	39.53 ± 3.01	<0.001*
CAR (mg/g)	0.283 ± 0.231	0.085 ± 0.075	<0.001*
CAR ≥ 0.1213 (n,%)	22(75.86)	76(25.33)	<0.001*
Procalcitonin (ng/mL)	0.079 ± 0.044	0.039 ± 0.019	<0.001*
ESR (mm/h)	38.76 ± 25.95	18.96 ± 9.99	<0.001*
WBC > 10*10^9^/L (n,%)	8(27.59)	30(10.00)	0.005*
Neutrophil > 6.3*10^9^/L (n,%)	6(20.69)	36(12.00)	0.181
Lymphocyte < 1.1*10^9^/L (n,%)	6(20.69)	46(15.33)	0.450
RBC < Lower limit (n,%)	6(20.69)	41(13.67)	0.302
Hemoglobin < Lower limit (n,%)	7(24.14)	47(15.67)	0.240
Platelet > 220*10^9^ (n,%)	17(58.62)	168(56.00)	0.786
FBG (mmol/L)	6.26 ± 3.02	5.83 ± 1.42	0.452

*P-value < 0.05.

### 3.3 Risk factors

**Figure 2:**
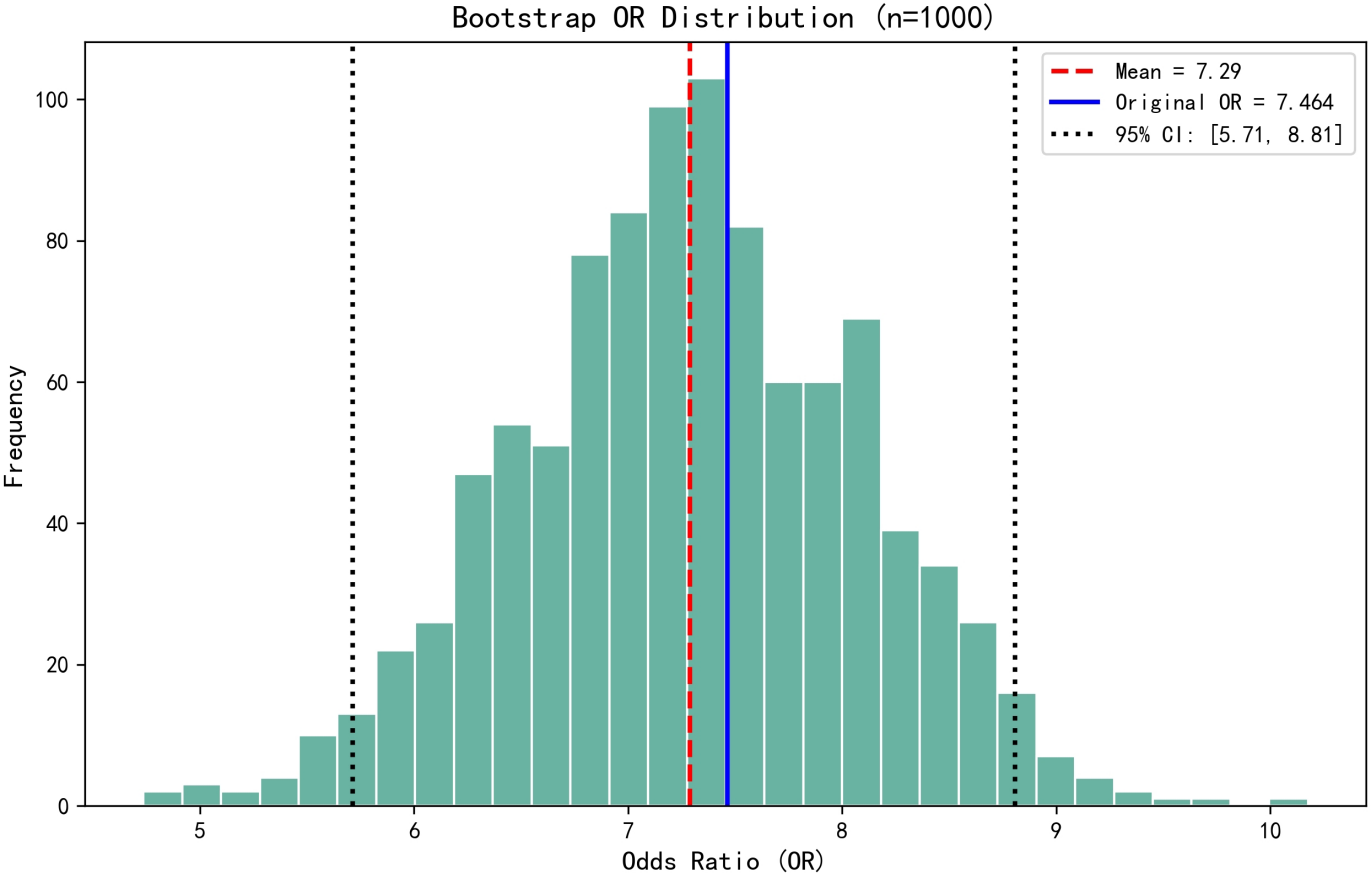
Bootstrap validation showed that the OR value of CAR remained stable over 1000 sampling times (mean OR = 7.29; 95% CI, 5.71–8.81), which was highly consistent with the original model (OR = 7.464).

**Figure 3:**
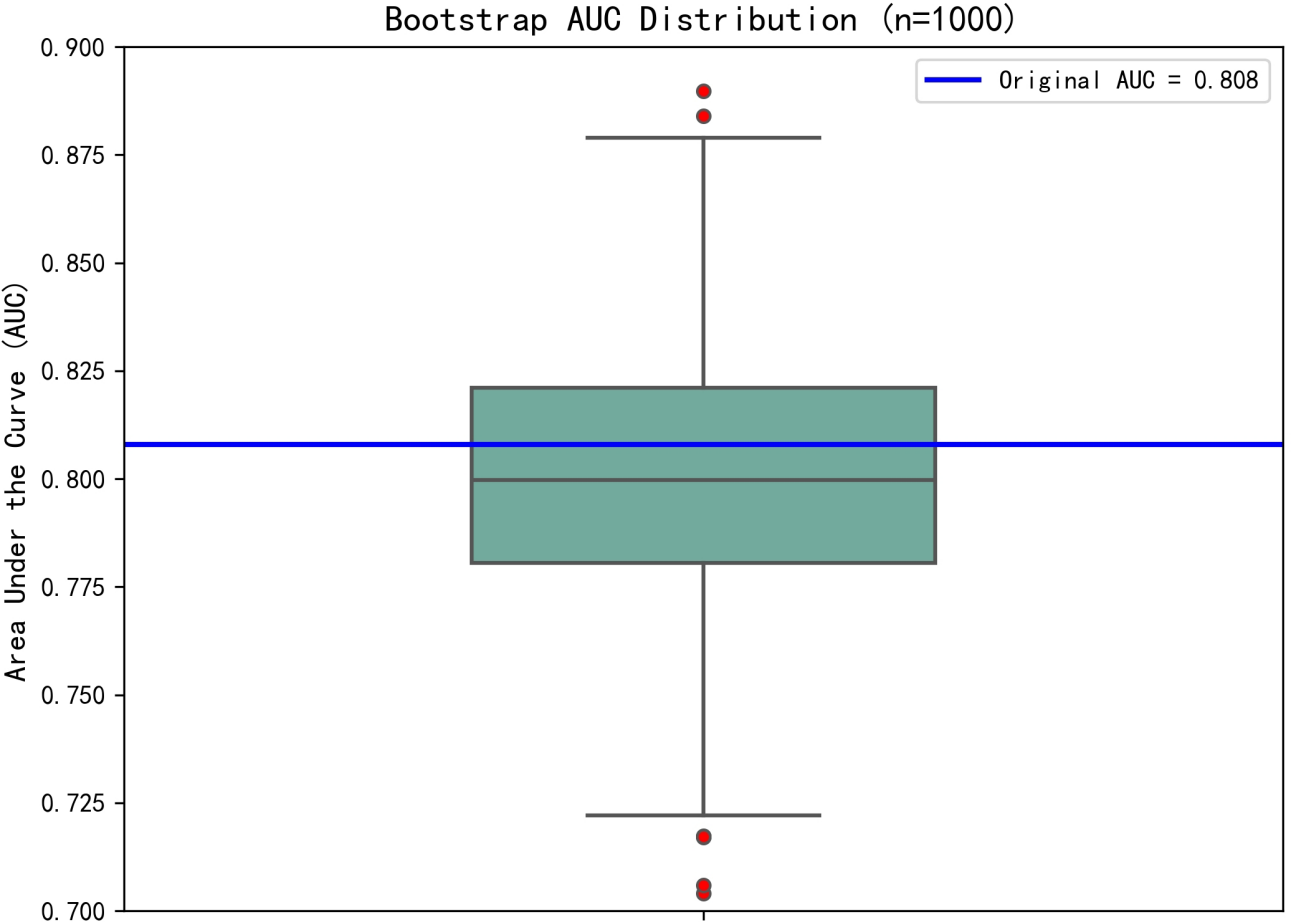
The Bootstrap mean value of the model prediction efficiency (AUC) was 0.801 (95% CI, 0.753–0.857), and the corrected AUC after overfitting correction was 0.797, indicating that the model had good stability.

The dichotomous logistic regression analyses revealed that CAR ≥0.1213 (OR = 7.464; 95% CI, 2.196–25.361; P < 0.001) was associated with 7.464-fold increased risk of SSI, together with the number of operated segments (OR = 3.825; 95% CI, 1.131–12.939; P = 0.031). Surgical duration (OR = 1.05; 95% CI, 1.027–1.073; P < 0.001) and ESR (OR = 1.046; 95% CI, 1.006–1.088; P = 0.023) were also independent risk factors associated with SSI. Serum albumin was identified as a protective factor for SSI (OR = 0.782; 95% CI, 0.664–0.920; P = 0.003). The results of the dichotomous logistic regression analyses are shown in **Table 3**. Bootstrap validation showed that the OR value of CAR remained stable over 1000 sampling times (mean OR = 7.29; 95% CI, 5.71–8.81), which was highly consistent with the original model (OR = 7.464) (**Figure 2**). The Bootstrap mean value of the model prediction efficiency (AUC) was 0.801 (95% CI, 0.753–0.857), and the corrected AUC after overfitting correction was 0.797, indicating that the model had good stability (**Figure 3**).

**Table 3:** Binary logistic regression analysis risk factors for SSI after PKP.

Variables	OR	95% CI	P-value
Surgical duration (minutes)	1.050	1.027-1.073	<0.001*
The number of operated segments	3.825	1.131-12.939	0.031*
Serum albumin (g/L)	0.782	0.664-0.920	0.003*
CAR ≥ 0.1213 (n,%)	7.464	2.196-25.361	<0.001*
ESR (mm/h)	1.046	1.006-1.088	0.023*

*P-value < 0.05.

### 3.4 Subgroup analysis

In the lumbar and thoracic subgroups, univariate regression analysis showed that the occurrence of SSI was also consistent with the variables related to SSI in **Table 2**, and all were significantly different (**Table 4**).

**Table 4:** Comparison between thoracic and lumbar subgroups.

Variables	Lumbar spine (n=109)			Thoracic vertebrae (n=220)		
	SSI Group (n=15)	Non-SSI Group (n=94)	P-value	SSI Group (n=14)	Non-SSI Group (n=206)	P-value
Sex (n,%)			0.354			0.573
Male	5(33.33)	21(22.34)		5(35.71)	59(28.64)	
Female	10(66.67)	88(77.66)		9(64.29)	147(71.36)	
Age (yr)	70.33 ± 6.24	67.77 ± 7.04	0.186	70.71 ± 6.62	68.92 ± 6.59)	0.324
BMI (kg/m^2^)	25.34 ± 2.78	24.66 ± 3.68	0.497	24.35 ± 1.83	24.34 ± 3.63	0.991
Hypertension (n,%)	7(46.67)	41(43.62	0.825	7(50.00)	82(39.81)	0.452
Diabetes mellitus (n,%)	2(13.33)	13(13.83)	0.959	2(14.29)	45(21.84)	0.504
Tobacco use (n,%)	0(0.00)	6(6.38)	0.314	1(7.14)	9(4.37)	0.630
Drinking history (n,%)	1(26.67)	6(6.38)	0.967	1(7.14)	6(2.91)	0.383
Surgical duration (minutes)	95.00 ± 41.75	51.17 ± 20.25	0.001*	84.29 ± 31.67	49.32 ± 18.79	0.001*
The number of operated segments	1.47 ± 0.52	1.13 ± 0.34	0.026*	1.71 ± 0.61	1.10 ± 0.33	0.002*
Preoperative body temperature ≥ 37.2°C (n,%)	4(26.67)	10(10.64)	0.085	2(14.29)	21(10.19)	0.628
Preoperative blood biochemical indicators						
CRP (mg/L)	10.15 ± 7.97	3.58 ± 2.74	0.007*	8.80 ± 6.02	3.25 ± 2.96	0.004*
Serum albumin (g/L)	35.39 ± 4.53	39.58 ± 2.91	0.003*	35.36 ± 4.94	39.50 ± 3.06	<0.001*
CAR (mg/g)	0.306 ± 0.268	0.090 ± 0.069	0.008*	0.259 ± 0.189	0.083 ± 0.078	0.004*
CAR ≥ 0.1213 (n,%)	11(73.33)	33(35.11)	0.005*	11(78.57)	43(20.87)	<0.001*
Procalcitonin (ng/mL)	0.082 ± 0.052	0.041 ± 0.018	0.009*	0.077 ± 0.036	0.039 ± 0.020	0.001*
ESR (mm/h)	37.73 ± 23.34)	19.80 ± 9.90	0.010*	39.86 ± 29.35	18.58 ± 10.03	0.018*
WBC > 10*10^9^/L (n,%)	4(26.67)	8(8.51)	0.037*	4(28.57)	22(10.68)	0.045*
Neutrophil > 6.3*10^9^/L (n,%)	4(26.67)	9(9.57)	0.058	2(14.29)	27(13.11)	0.900
Lymphocyte < 1.1*10^9^/L (n,%)	4(26.67)	14(14.89)	0.254	2(14.29)	32(15.53)	0.900
RBC < Lower limit (n,%)	3(20.00)	14(14.89)	0.613	3(21.43)	27(13.11)	0.380
Hemoglobin < Lower limit (n,%)	2(13.33)	14(14.89)	0.874	5(35.71)	33(16.02)	0.059
Platelet > 220*10^9^ (n,%)	6(40.00)	52(55.32)	0.269	11(78.57)	116(56.31)	0.103
FBG (mmol/L)	7.20 ± 3.96	5.67 ± 1.14	0.160	5.25 ± 0.80	5.90 ± 1.53	0.120

*P-value < 0.05.

Binary logistic regression analysis of subgroups showed that surgical duration (OR = 1.063; 95% CI, 1.029–1.098; P < 0.001) and preoperative CRP (OR = 1.409; 95% CI, 1.043–1.903; P = 0.026) were independent risk factors associated with SSI in the lumbar spine subgroup. Meanwhile, serum albumin was identified as a protective factor for SSI (OR = 0.665; 95% CI, 0.481–0.921; P = 0.014). In addition, surgical duration (OR = 1.044; 95% CI, 1.009–1.079; P = 0.013), the number of operated segments (OR = 8.561; 95% CI, 2.005–36.551; P = 0.004), CAR ≥0.1213 (OR = 8.861; 95% CI, 1.603–48.975; P = 0.012), ESR (OR = 1.046; 95% CI, 1.005–1.088; P = 0.028) and WBC > 10*109/L (OR = 6.005; 95% CI, 1.042–34.628; P = 0.045) were independent risk factors for SSI in the thoracic vertebrae subgroup (**Table 5**).

**Table 5:** Binary logistic regression analysis risk factors for SSI after PKP in subgroups.

Variables	Lumbar spine			Thoracic vertebrae		
	OR	95% CI	P-value	OR	95% CI	P-value
Surgical duration (minutes)	1.063	1.029-1.098	<0.001*	1.044	1.009-1.079	0.013*
The number of operated segments	–	–	–	8.561	2.005-36.551	0.004*
Serum albumin (g/L)	0.665	0.481-0.921	0.014*	–	–	–
CAR ≥ 0.1213 (n,%)	–	–	–	8.861	1.603-48.975	0.012*
ESR (mm/h)	–	–	–	1.046	1.005-1.088	0.028*
CRP (mg/L)	1.409	1.043-1.903	0.026*	–	–	–
WBC > 10*10^9^/L (n,%)	–	–	–	6.005	1.042-34.628	0.045*

*P-value < 0.05.

## 4 Discussion

This study has some notable findings: preoperative CAR can predict the occurrence of post-PKP SSI for thoracolumbar compression fractures with a large correlation (OR = 7.464). Serum albumin is a protective factor for SSI after PKP. In addition, we found that the more surgical segments, the higher the risk of post-PKP SSI (1.59 ± 0.57 vs 1.11 ± 0.33, P < 0.001). More surgical segments indicate longer operation time, more blood loss, and more fluoroscopy times, which are high-risk factors for postoperative SSI (Meng et al., 2015; Ogihara et al., 2015).

Device implantation surgery is a major factor contributing to SSI because the interface formed between the implant and human tissue provides a favorable site for bacterial colonization. At this interface, bacteria can form a biofilm, which is a complex protective matrix composed of proteins, extracellular polysaccharides, and extracellular DNA. This biofilm shields bacteria from attacks by the host immune system and the effects of antibiotics, thereby increasing the risk of infection (Bouvresse et al., 2006; Abdelrahman et al., 2013; Jia-Jia et al., 2018; Park et al., 2018). Evidently, this stands in direct opposition to the principle of PKP, which requires the uneven and extensive dispersion of bone cement within the affected vertebra (Pan et al., 2022). Furthermore, once an infection occurs, antibiotic therapy alone is often insufficient for complete eradication. Revision surgery to remove the bone cement can cause significant trauma and lead to severe complications. Therefore, establishing effective predictive methods for SSI after PKP and implementing early interventions are crucial.

CRP is an acute-phase protein, and in 1930, Tillett and Francis identified proteins responsive to the systemic C-polysaccharide response of Streptococcus pneumoniae in patients with acute pneumococcal infection (Seok and Ju, 2020), and it was later named CRP. CRP is produced in the liver and regulated by several proinflammatory cytokines, such as interleukin-1, interleukin-6, and tumor necrosis factor-α (Rizo-Téllez et al., 2023). Healthy individuals have low levels of serum CRP, which usually increases in various inflammation, infection, stress, trauma, surgery, and tissue injury.

Serum albumin is an essential protein synthesized by hepatocytes. It not only plays a crucial role in regulating plasma osmotic pressure but also serves as a transporter for various substances involved in acute and chronic inflammatory processes (Kayapinar et al., 2019). Its levels are determined by subtle changes in hepatic synthesis and catabolism, reflecting the immune and nutritional status of the body. In infection or chronic inflammation, serum albumin levels decrease. Conversely, a reduction in serum albumin also indicates a decline in immune function, rendering the body less resistant to pathogens and more susceptible to infections (Sheinenzon et al., 2021).

However, CRP is a nonspecific marker of inflammation, that is, it is not only elevated in response to infection. A variety of other conditions, such as inflammatory diseases, autoimmune diseases, tumors, and cardiovascular diseases, may lead to elevated CRP levels (Orr et al., 2018; Mouliou, 2023). False-negative results may occur when its serum concentration is low (Zimmerli and Moser, 2012). In addition, owing to the relatively long half-life of serum albumin, its levels do not exhibit a clear correlation with the body’s condition before infection or the acute inflammatory response (Shi et al., 2021). Therefore, when CRP or serum albumin is used independently as a preoperative predictor of postoperative SSI, the results are often unsatisfactory.

CAR, as a novel indicator, combines the advantages of CRP and serum albumin. It reflects not only the inflammatory status of the body but also its nutritional status (Kaplan et al., 2017). Thus, CAR demonstrates better predictive performance than single variables, reducing the errors associated with CRP or albumin alone (Güneş et al., 2021). Currently, several studies have validated CAR as a useful prognostic indicator for conditions such as myocardial infarction, cancer, or emergency abdominal surgery (Simpson et al., 2018; Askin et al., 2020; Alkurt et al., 2022). In summary, CAR can serve as a more reliable biomarker for predicting the severity and prognosis of various diseases.

However, the optimal cut-off value selected according to the ROC curve is often inconsistent or even very different in predicting the risk of developing different diseases or complications. For example, Konishi et al (Konishi et al., 2019) used 0.05 as the optimal cut-off value to predict overall survival in metastatic renal cell carcinoma, whereas Capkin et al (Capkin et al., 2021) selected 2.49 as the optimal cut-off value to predict mortality for elderly population who undergo hemiarthroplasty due to hip fracture. In addition, the prediction of disease by preoperative CAR does not necessarily apply to other different types of diseases, and some researchers have suggested that early postoperative CAR rather than preoperative CAR is more applicable (Donlon et al., 2020). For example, Liu et al (Liu et al., 2022) proposed using the CAR on postoperative day 1 to predict short-term complications of gastric cancer surgery.

In the present study, we determined the optimal cut‐off of CAR for predicting SSI after PKP to be 0.1213, equal to or higher than which was associated with 7.464‐fold increased risk of SSI. The corresponding sensitivity, specificity, and AUC were 0.759, 0.747, and 0.808, respectively. The strong correlation between a high CAR and SSI suggests that it has the potential to become an effective predictive marker for postoperative infections following PKP. Surgeons can use this value to make informed clinical decisions, such as delaying surgery, implementing prophylactic anti-infective measures, or adjusting treatment plans, thereby keeping the risk of SSI at a relatively low level.

During the course of the study, according to our data, the incidence of SSI after PKP in lumbar compression fractures (13.8%) was higher than that in thoracic vertebrae (6.4%). We also reviewed the literature that spinal surgery close to the lumbosacral region is a high-risk factor for increasing SSI after spinal surgery (Abdul-Jabbar et al., 2012). The volume of lumbar vertebral body is larger than that of thoracic vertebral body (Limthongkul et al., 2010), and the amount of bone cement injected into lumbar vertebral body is greater than that of thoracic vertebral body during operation. For this purpose, we also performed analyses of the lumbar and thoracic subgroups. The results showed that in the lumbar spine subgroup, compared with the total analysis, preoperative CRP was an independent risk factor for SSI after lumbar vertebral compression fracture surgery. In the thoracic subgroup, WBC > 10*109/L was also a higher risk factor for postoperative SSI. WBC is also of some value in predicting certain infectious diseases (Tascini et al., 2020).

## 5 Limitations

It must be noted that this study has some limitations. First of all, the inherent limitations of retrospective studies in data collection, such as data integrity, quality, bias and time sensitivity, and the confounding effects of some unmeasured or unknown factors in binary logistic regression analysis, will negatively affect the accuracy of research results. While our exclusion criteria removed patients with active infections or recent antibiotic use, retrospective data limited our ability to account for variations in preoperative antibiotic regimens. Future prospective studies should standardize and document antibiotic protocols to minimize this bias. Secondly, individual differences in patients, nutritional status, coexisting diseases, and other factors may affect the levels of CRP and serum albumin, thereby affecting the interpretation of CAR. Finally, the number of patients included in this study was relatively small, and the single-center design compromises the generalizability of the findings to other settings. Therefore, further large sample epidemiological data and more comprehensive laboratory indicators are needed for verification, and prospective multicenter studies are needed to verify the generalization of the conclusions.

## 6 Conclusions

In conclusion, our study demonstrated that preoperative CAR was independently associated with SSI after PKP in thoracolumbar compression fractures, with a 7.464-fold increased risk. Future studies should further validate its validity and consider it as a useful routine tool for predicting the occurrence of SSI after PKP.

## Data Availability

The raw data supporting the conclusions of this article will be made available by the authors, without undue reservation.

## References

[B1] Abdelrahman H. Siam A. E. Shawky A. Ezzati A. Boehm H. (2013). Infection after vertebroplasty or kyphoplasty. A Ser. nine cases Rev. literature. Spine J. 13, 1809–1817. doi: 10.1016/j.spinee.2013.05.053 23880354

[B2] Abdul-Jabbar A. Takemoto S. Weber M. H. Hu S. S. Mummaneni P. V. Deviren V.. (2012). Surgical site infection in spinal surgery: description of surgical and patient-based risk factors for postoperative infection using administrative claims data. Spine. 37, 1340–1345. doi: 10.1097/BRS.0b013e318246a53a 22210012

[B3] Alkurt E. G. Durak D. Turhan V. B. Sahiner I. T. (2022). Effect of C-reactive protein-to-albumin ratio on prognosis in gastric cancer patients. Cureus. 14, e23972. doi: 10.7759/cureus.23972 35547460 PMC9090126

[B4] Askin L. Tanriverdi O. Tibilli H. Turkmen S. (2020). Prognostic value of C-reactive protein/albumin ratio in ST-segment elevation myocardial infarction. Interventional Med. Appl. science. 11, 168–171. doi: 10.1556/1646.11.2019.20 PMC9467334 36343286

[B5] Berríos-Torres S. I. Umscheid C. A. Bratzler D. W. Leas B. Stone E. C. Kelz R. R.. (2017). Centers for disease control and prevention guideline for the prevention of surgical site infection, 2017. JAMA surgery. 152, 784–791. doi: 10.1001/jamasurg.2017.0904 28467526

[B6] Boonen S. Wahl D. A. Nauroy L. Brandi M. L. Bouxsein M. L. Goldhahn J.. (2011). Balloon kyphoplasty and vertebroplasty in the management of vertebral compression fractures. Osteoporosis Int. 22, 2915–2934. doi: 10.1007/s00198-011-1639-5 21789685

[B7] Bouvresse S. Chiras J. Bricaire F. Bossi P. (2006). Pott’s disease occurring after percutaneous vertebroplasty: an unusual illustration of the principle of locus minoris resistentiae. J. infection. 53, e251–e253. doi: 10.1016/j.jinf.2006.02.014 16584785

[B8] Capkin S. Guler S. Ozmanevra R. (2021). C-reactive protein to albumin ratio may predict mortality for elderly population who undergo hemiarthroplasty due to hip fracture. J. Invest. Surg. 34, 1272–1277. doi: 10.1080/08941939.2020.1793038 32668996

[B9] Donlon N. E. Mohan H. Free R. Elbaghir B. Soric I. Fleming C.. (2020). Predictive value of CRP/albumin ratio in major abdominal surgery. Irish J. Med. science. 189, 1465–1470. doi: 10.1007/s11845-020-02238-y 32361882

[B10] Goulart A. Ferreira C. Estrada A. Nogueira F. Martins S. Mesquita-Rodrigues A.. (2018). Early inflammatory biomarkers as predictive factors for freedom from infection after colorectal cancer surgery: A prospective cohort study. Surg. infections. 19, 446–450. doi: 10.1089/sur.2017.294 29624484

[B11] Güneş H. Yurttutan S. Çobanuşağı M. Doğaner A. (2021). CRP/albumin ratio: A promising marker of gram-negative bacteremia in late-onset neonatal sepsis. Turkish Arch. pediatrics. 56, 32–36. doi: 10.14744/TurkPediatriArs.2020.99076 PMC8114609 34013227

[B12] Harrell F. E. (2001). Regression modeling strategies: with applications to linear models, logistic regression, and survival analysis (New York, NY: Springer).

[B13] Iasonos A. Schrag D. Raj G. V. Panageas K. S. (2008). How to build and interpret a nomogram for cancer prognosis. J. Clin. Oncol. 26, 1364–1370. doi: 10.1200/jco.2007.12.9791 18323559

[B14] Jia-Jia S. Zhi-Yong S. Zhong-Lai Q. Hui-Lin Y. Xiao-Yu Z. (2018). Tuberculous spondylitis after vertebral augmentation: A case report with a literature review. J. Int. Med. Res. 46, 916–924. doi: 10.1177/0300060517728008 29239241 PMC5971511

[B15] Jia-Min Z. Wei D. Ye L. Xiang-Tao P. (2022). Correlation between C-reactive protein/albumin ratio and prognosis in patients with lung adenocarcinoma. J. Int. Med. Res. 50, 3000605221105372. doi: 10.1177/03000605221105372 35730330 PMC9228648

[B16] Kaplan M. Ates I. Akpinar M. Y. Yuksel M. Kuzu U. B. Kacar S.. (2017). Predictive value of C-reactive protein/albumin ratio in acute pancreatitis. Hepatobiliary pancreatic Dis. international: HBPD Int. 16, 424–430. doi: 10.1016/s1499-3872(17)60007-9 28823374

[B17] Kayapinar O. Ozde C. Kaya A. (2019). Relationship between the reciprocal change in inflammation-related biomarkers (Fibrinogen-to-albumin and hsCRP-to-albumin ratios) and the presence and severity of coronary slow flow. Clin. Appl. thrombosis/hemostasis 25, 1076029619835383. doi: 10.1177/1076029619835383 PMC6714912 30857397

[B18] Konishi S. Hatakeyama S. Tanaka T. Ikehata Y. Tanaka T. Hamano I.. (2019). C-reactive protein/albumin ratio is a predictive factor for prognosis in patients with metastatic renal cell carcinoma. Int. J. Urol. 26, 992–998. doi: 10.1111/iju.v26.10 31342557

[B19] Li Y. J. Yao K. Lu M. X. Zhang W. B. Xiao C. Tu C. Q. (2017). Prognostic value of the C-reactive protein to albumin ratio: a novel inflammation-based prognostic indicator in osteosarcoma. OncoTargets Ther. 10, 5255–5261. doi: 10.2147/OTT.S140560 PMC5679688 29138578

[B20] Liao M. Xie Y. Mao Y. Lu Z. Tan A. Wu C.. (2018). Comparative analyses of fecal microbiota in Chinese isolated Yao population, minority Zhuang and rural Han by 16sRNA sequencing. Sci. Rep. 8, 1142. doi: 10.1038/s41598-017-17851-8 29348587 PMC5773753

[B21] Limthongkul W. Karaikovic E. E. Savage J. W. Markovic A. (2010). Volumetric analysis of thoracic and lumbar vertebral bodies. Spine J. 10, 153–158. doi: 10.1016/j.spinee.2009.11.018 20142072

[B22] Liu Z. Chen L. Sun F. Lv B. Ge X. Shao L.. (2022). C-reactive protein/albumin ratio on the first day after surgery predicts short-term complications of gastrectomy for gastric cancer. Nutr. cancer. 74, 3574–3581. doi: 10.1080/01635581.2022.2083190 35762207

[B23] Mai R. Y. Lu T. L. Lu R. J. Zeng C. Lian F. Li L. Q.. (2024). C-reactive protein-albumin ratio (CAR): A more promising inflammation-based prognostic marker for patients undergoing curative hepatectomy for hepatocellular carcinoma. J. Inflammation Res. 17, 919–931. doi: 10.2147/JIR.S441623 PMC10871143 38370468

[B24] Meng F. Cao J. Meng X. (2015). Risk factors for surgical site infections following spinal surgery. J. Clin. Neurosci. 22, 1862–1866. doi: 10.1016/j.jocn.2015.03.065 26282155

[B25] Mouliou D. S. (2023). C-reactive protein: pathophysiology, diagnosis, false test results and a novel diagnostic algorithm for clinicians. Dis. (Basel Switzerland) 11, 132. doi: 10.3390/diseases11040132 PMC10594506 37873776

[B26] Ning P. Yang B. Yang X. Huang H. Shen Q. Zhao Q.. (2021). Clinical value of C-reactive protein/albumin ratio in Guillain-Barré syndrome. Neurological Sci. 42, 3275–3283. doi: 10.1007/s10072-020-04930-4 33247320

[B27] Oei L. Koromani F. Breda S. J. Schousboe J. T. Clark E. M. van Meurs J. B.. (2018). Osteoporotic vertebral fracture prevalence varies widely between qualitative and quantitative radiological assessment methods: the rotterdam study. J. Bone mineral Res. 33, 560–568. doi: 10.1002/jbmr.3220 28719143

[B28] Ogihara S. Yamazaki T. Maruyama T. Oka H. Miyoshi K. Azuma S.. (2015). Prospective multicenter surveillance and risk factor analysis of deep surgical site infection after posterior thoracic and/or lumbar spinal surgery in adults. J. orthopaedic Sci. 20, 71–77. doi: 10.1007/s00776-014-0669-1 25366698

[B29] Orr C. K. Najm A. Young F. McGarry T. Biniecka M. Fearon U.. (2018). The utility and limitations of CRP, ESR and DAS28-CRP in appraising disease activity in rheumatoid arthritis. Front. Med. 5, 185. doi: 10.3389/fmed.2018.00185 PMC6085449 30123796

[B30] Pamukcu M. Duran T. I. (2021). Could C-reactive protein/albumin ratio be an indicator of activation in axial spondyloarthritis? J. Coll. Physicians Surgeons–Pakistan: JCPSP 31, 537–541. doi: 10.29271/jcpsp.2021.05.537 34027865

[B31] Pan Z. Zhou Q. Yang M. Deng L. Hu X. Lv N.. (2022). Effects of distribution of bone cement on clinical efficacy and secondary fracture after percutaneous kyphoplasty for osteoporotic vertebral compression fractures. Front. surgery. 9, 1054995. doi: 10.3389/fsurg.2022.1054995 PMC9852057 36684222

[B32] Papanastassiou I. D. Phillips F. M. Van Meirhaeghe J. Berenson J. R. Andersson G. B. Chung G.. (2012). Comparing effects of kyphoplasty, vertebroplasty, and non-surgical management in a systematic review of randomized and non-randomized controlled studies. Eur. Spine J. 21, 1826–1843. doi: 10.1007/s00586-012-2314-z 22543412 PMC3459114

[B33] Park J. W. Park S. M. Lee H. J. Lee C. K. Chang B. S. Kim H. (2018). Infection following percutaneous vertebral augmentation with polymethylmethacrylate. Arch. osteoporosis. 13, 47. doi: 10.1007/s11657-018-0468-y 29704173

[B34] Ren Y. Fan X. Chen G. Zhou D. Lin H. Cai X. (2019). Preoperative C-reactive protein/albumin ratio to predict mortality and recurrence of patients with hepatocellular carcinoma after curative resection. Medicina clinica. 153, 183–190. doi: 10.1016/j.medcli.2018.11.010 30606506

[B35] Rizo-Téllez S. A. Sekheri M. Filep J. G. (2023). C-reactive protein: a target for therapy to reduce inflammation. Front. Immunol. 14, 1237729. doi: 10.3389/fimmu.2023.1237729 37564640 PMC10410079

[B36] Seok J. S. Ju H. (2020). Plasmonic optical biosensors for detecting C-reactive protein: A review. Micromachines. 11 (10), 895. doi: 10.3390/mi11100895 32992442 PMC7599671

[B37] Sheinenzon A. Shehadeh M. Michelis R. Shaoul E. Ronen O. (2021). Serum albumin levels and inflammation. Int. J. Biol. macromolecules. 184, 857–862. doi: 10.1016/j.ijbiomac.2021.06.140 34181998

[B38] Shi W. Wang Y. Zhao X. Yu T. Li T. (2021). CRP/albumin has a promising prospect as a new biomarker for the diagnosis of periprosthetic joint infection. Infection Drug resistance. 14, 5145–5151. doi: 10.2147/IDR.S342652 34908848 PMC8664647

[B39] Simpson G. Saunders R. Wilson J. Magee C. (2018). The role of the neutrophil:lymphocyte ratio (NLR) and the CRP:albumin ratio (CAR) in predicting mortality following emergency laparotomy in the over 80 age group. Eur. J. Trauma Emergency Surg. 44, 877–882. doi: 10.1007/s00068-017-0869-4 29134253

[B40] Tascini C. Aimo A. Arzilli C. Sbrana F. Ripoli A. Ghiadoni L.. (2020). Procalcitonin, white blood cell count and C-reactive protein as predictors of S. aureus infection and mortality in infective endocarditis. Int. J. Cardiol. 301, 190–194. doi: 10.1016/j.ijcard.2019.08.013 31405585

[B41] Uchimoto T. Matsuda T. Komura K. Fukuokaya W. Adachi T. Hirasawa Y.. (2024). C-reactive protein-albumin ratio predicts objective response to enfortumab vedotin in metastatic urothelial carcinoma. Targeted Oncol. 19, 635–644. doi: 10.1007/s11523-024-01068-7 38807017

[B42] Wu Y. Sun K. Liu R. Wu L. Zeng Y. Li M.. (2023). C-reactive protein/albumin and C-reactive protein/fibrinogen ratios for the diagnosis of periprosthetic joint infection in revision total joint arthroplasty. Int. immunopharmacology. 115, 109682. doi: 10.1016/j.intimp.2023.109682 36623413

[B43] Zhu H. T. Ding D. G. Wang S. Zhu Y. L. (2022). Comparison between percutaneous kyphoplasty and percutaneous vertebroplasty in terms of efficacy in osteoporotic vertebral compression fractures: A meta-analysis. Altern. therapies Health Med. 28, 49–53. doi: 10.21203/rs.3.rs-609136/v1 35648693

[B44] Ziegler M. A. Bauman J. C. Welsh R. J. Wasvary H. J. (2022). Can the American college of surgeons national surgical quality improvement program risk calculator predict outcomes for urgent colectomies? Am. surgeon 88, 65–69. doi: 10.1177/0003134820973392 33345578

[B45] Zimmerli W. Moser C. (2012). Pathogenesis and treatment concepts of orthopaedic biofilm infections. FEMS Immunol. Med. Microbiol. 65, 158–168. doi: 10.1111/j.1574-695X.2012.00938.x 22309166

